# FgMet3 and FgMet14 related to cysteine and methionine biosynthesis regulate vegetative growth, sexual reproduction, pathogenicity, and sensitivity to fungicides in *Fusarium graminearum*

**DOI:** 10.3389/fpls.2022.1011709

**Published:** 2022-10-24

**Authors:** Feifei Zhao, Zhili Yuan, Weidong Wen, Zhongyu Huang, Xuewei Mao, Mingguo Zhou, Yiping Hou

**Affiliations:** College of Plant Protection, Nanjing Agricultural University, Jiangsu, China

**Keywords:** *fusarium graminearum*, cysteine, methionine, FgMet3, FgMet14, sexual reproduction, pathogenicity, fungicide sensitivity

## Abstract

*Fusarium graminearum* is a destructive filamentous fungus, which widely exists in wheat and other cereal crops. Cysteine and Methionine are unique sulfur-containing amino acids that play an essential role in protein synthesis and cell life, but their functions and regulation in *F. graminearum* remain largely unknown. Here we identified two proteins, FgMet3 and FgMet14 in *F. graminearum*, which are related to the synthesis of cysteine and methionine. We found FgMet3 and FgMet14 were localized to the cytoplasm and there was an interaction between them. *FgMet3* or *FgMet14* deletion mutants (Δ*FgMet3* and Δ*FgMet14*) were deficient in vegetative growth, pigment formation, sexual development, penetrability and pathogenicity. With exogenous addition of cysteine and methionine, the vegetative growth and penetrability could be completely restored in Δ*FgMet3* and Δ*FgMet14*, while sexual reproduction could be fully restored in Δ*FgMet3* and partially restored in Δ*FgMet14*. Δ*FgMet3* and Δ*FgMet14* exhibited decreased sensitivity to Congo red stress and increased sensitivity to SDS, NaCl, KCl, Sorbitol, Menadione, and Zn ion stresses. Moreover, FgMet3 and FgMet14 nonspecifically regulate the sensitivity of *F. graminearum* to fungicides. In conclusion, FgMet3 and FgMet14 interacted to jointly regulate the development, pathogenicity, pigment formation, sensitivity to fungicides and stress factors in *F. graminearum*.

## Introduction

*F.graminearum* is the main pathogen of wheat scab (Fusarium head blight, FHB) (Bechtel et al., 1985; Miedaner and Talas, 2011). It is a destructive filamentous fungus, which widely exists in wheat and other cereal crops (Dal Bello et al., 2002; Krstanovic et al., 2003; Erayman et al., 2015). *F. graminearum* occurs worldwide, especially in humid and rainy temperate regions. Fusarium head blight caused by *F. graminearum* not only reduces wheat yield (Lu and Faris, 2019), its pathogen also produces mycotoxins such as deoxynivalenol (DON) and zearalenone (ZEN) (Zhang et al., 2019; Xu et al., 2021; Zhou et al., 2021). If the wheat and its products contaminated by mycotoxins are eaten by humans and animals, they will cause vomiting, diarrhea, abortion, stillbirth, and other problems, which pose a great threat to the health of human beings and animals (Puri and Zhong, 2010; Zheng et al., 2021). A comprehensive understanding of the molecular mechanism related to fungal growth, development, and pathogenicity is helpful to formulate effective measures to control this destructive pathogen and provide guidance for the development of new and efficient fungicides (Becher et al., 2010; Chen et al., 2020).

Cysteine (Cys) and methionine (Met) are two unique sulfur-containing amino acids which play an important role in cell life activities (Gai et al., 2021). Cysteine is a highly conserved amino acid residue in proteins (Khamcharoen et al., 2022). Cysteine has a variety of functions, including the regulation of catalysis, structure, redox sensitivity, and metal transport (Wang et al., 2021; Wen et al., 2021). Importantly, cysteine is highly ionized and has oxidized sensitive sulfur atoms, responding to different pH and redox conditions of eukaryotic subcellular organelles (Ghaffari et al., 2020; Wen et al., 2021). Methionine, as a free amino acid, can be oxidized to methionine sulfoxide, which plays an important role in protecting organisms from oxidative stress (Catanesi et al., 2021; Fang et al., 2021). In *Saccharomyces cerevisiae*, inorganic sulfate is mainly used as the precursor of cysteine and methionine biosynthesis. After inorganic sulfate enters the cell, it is activated by ATP sulfase encoded by Met3 to produce adenosine-5′-phosphosulfate (APS) (Li et al., 2020). Aps is then activated by aps kinases encoded by Met14 to produce 3′-phosphoadenosine-5′-phosphosulfate (PAPS) (Li et al., 2020). The sulfite is then reduced to sulfide by sulfite reductase to synthesize homocysteine, cysteine, and methionine in the organism (Li et al., 2020). Studies have shown that amino acids are of great significance to the growth and development of fungi. However, the regulation of cysteine and methionine in *F. graminearum* remain unclear now.

In this study, we explored the functions and regulation of FgMet3 and FgMet14 which related to cysteine and methionine biosynthesis in *F. graminearum*. The results revealed that FgMet3 interacted with FgMet14, and they regulated vegetative growth, pigment formation, sexual development, penetrability and pathogenicity, sensitivity to fungicides, and response to different stress factors in *F. graminearum*. Our study provided a certain guiding significance for the comprehensive control of wheat scab.

## Materials and methods

### Strains, culture conditions, and fungicides

The wild-type strain PH-1 of *Fusarium graminearum* was preserved in the laboratory, and other mutants were derived from PH-1. All strains were cultured on PDA medium (200 g potato, 20 g glucose, and 16 g agar/L) at 25°C.

In order to determine the sensitivity of strains to different fungicides, phenamacril (95%, Jiangsu Pesticide Research Institute Co., Ltd.), fluoxastrobin (94%, Bayer Co., Ltd.), pydiflumetofen (98%, Swiss Syngenta crop Protection Co., Ltd.), tebuconazole (98%, Jiangsu Dragon Lantern Chemical Co., Ltd.), carbendazim (98%, Jiangsu Dragon Lantern Chemical Co., Ltd.), cyprodinil (98%, Swiss Syngenta crop Protection Co., Ltd.), chlorothalonil (98%, Swiss Syngenta crop Protection Co., Ltd.), fludioxonil (95%, Swiss Syngenta crop Protection Co., Ltd.), prothioconazole (95%, Shandong Hailier Chemical Co., Ltd.), fluazinam (98%, Nanjing Red Sun Co., Ltd) were used in this experiment.

### Generation of deletion mutants, GFP and 3×FLAG fusion strains

*FgMet3* deletion mutant was obtained by using homologous recombination (**Figure S1**). The upstream fragment of FgMet3 about 1kb and the downstream fragment of about 1kb were amplified by primer pair P1/P2 and P3/P4, and the HPH-Hsv-tk fragment was amplified by primer pair P5/P6, then the upstream and downstream fragments and HPH-Hsv-tk fragment were fused by PCR fusion program (Yu et al., 2004). Protoplast preparation and transformation was performed as previously reported (Hou et al., 2002). Then total DNA of transformants was extracted (Bian et al., 2021) and verified by PCR and Sothern blot (**Figure S1**) (Yun et al., 2020). The HPH-Hsv-tk fragment in *FgMet3* deletion mutant was replaced by the full-length fragment containing the upstream and downstream of *FgMet3* to obtain Δ*FgMet3*-C strain. Δ*FgMet14*, Δ*FgMet14*-C and FgMet3-3×FLAG strains were also obtained by this method. All the primers used are shown in **Table S1**.

In order to obtain the FgMet3-GFP and FgMet14-GFP strains, the FgMet3-GFP and FgMet14-GFP vectors was constructed. The construction method is as described before (Tang et al., 2020). The strains were verified by western blot test.

### Vegetative growth and conidiation assays

For the vegetative growth rate experiment, all strains were transferred to PDA plate and cultured at 25°C for 3 days. 5 mm fresh plates on the edge of PDA plate were transferred to potato dextrose agar (PDA), minimal medium (MM), complete medium (CM) (Tang et al., 2020) and V8 juice medium (Janevska et al., 2018) culture plates with three repeats per treatment for 3 days. The cross-crossing method was adopted to measure the colony diameter.


Mycelial growth rate (mm/day)=(average mycelial diameter−5)/3

For conidiation assay, a fresh 5 mm plate of the strain was transferred to Mung bean liquid (MBL) medium (30 g mung beans/L) and cultured at 25°C under 175 rpm light for 3 days. The spore production of the strain was determined by the blood counting chamber. The 100 μL conidia suspension of 10^6^/mL was coated on Water Agar (WA) medium (16 g agar/L), and the germination rate of conidia was determined at 4 h and 8 h, respectively. The experiment was repeated three times.

### The sensitivity of strains to different fungicides and stress factors

For fungicides sensitivity assay, fresh 5 mm dishes taken from the edge of colonies on PDA plates were transferred to MM plates containing 0.5 μg/mL carbendazim, 0.03125 μg/mL fludioxonil, 0.2 μg/mL tebuconazole, 0.01 μg/mL pydiflumetofen, 4 μg/mL cyprodinil, 0.1 μg/mL fluoxastrobin, 0.2 μg/mL phenamacril, 0.4 μg/mL chlorothalonil, 0.5 μg/mL prothioconazole, 0.1 μg/mL fluazinam respectively. Three repeats in each treatment were cultured at 25°C for 3 days, and the colony diameter was measured by cross method. The experiment was repeated three times.

For stresses sensitivity assay, all strains were transferred to PDA plates and cultured at 25°C for 3 d. Fresh 5 mm dishes were taken from the edge of colonies and transferred to PDA plates containing 500 µg/mL Congo red, 0.03% SDS, 1 M NaCl, 1 M KCl, 1.5 M Sorbitol, 0.1 mM Menadione, 5 mM ZnCl_2_, and 0.4 M CaCl_2_ (Chen et al., 2018). There were three repeats in each treatment and cultured at 25°C for 3 days. The colony diameter was measured by the cross-crossing method, and the growth inhibition rate of mycelia was calculated. The experiment was repeated for three times.


Mycelial growth inhibition rate(%)=(average mycelial diameter of the control group−average mycelial diameter of each experimental group)/(average mycelial diameter of the control group−5)×100.

### Sexual reproduction assay

For sexual reproduction, the strains were cultured on carrot medium for 4 days, scraped the hyphae on Carrot medium with 2.5% Tween -20, and then cultured in 25°C under a near-UV light (Shin et al., 2017; Bian et al., 2021). On the 7th, 11th, and 15th day of culture, the perithecia and the ascospores were observed with a Nikon SMZ25 fluorescence stereomicroscope and an Olympus IX-71 inverted fluorescence microscope. In order to determine whether the strain has the ability to eject ascospores, the plate was made on carrot medium with a punch on the 7th, 11th, and 15th day, and the plate was cut in half with a sterilized scalpel. The cut side was placed on a glass slide and cultured at 25°C for 24-48 hours, then observed with a Nikon SMZ25 fluorescence stereomicroscope. The experiment was repeated three times.

### Pathogenicity and penetration assays

After the strain was shaken in MBL medium for 3 days, the filtrate was filtered with three layers of scrubbing paper, and the conidia were obtained by 5000 rpm centrifugation of 5 mins and washed 2-3 times with sterilized distilled water at the same rotational speed. Finally, an appropriate amount of distilled water was added to make the concentration of 10^6^ conidia/mL. The pathogenicity assay on the wheat heads was conducted by injecting 10 μL conidial suspension into a floret in the central section spikelet of the susceptible cultivar Huaimai 33 in the field, and 10 μL distilled water was inoculated as control (Kong et al., 2018). The incidence of the wheat spike was investigated after being cultured at 25°C for 14 days. For the pathogenicity experiment of wheat coleoptile, the wound was made on wheat coleoptile, and 2 μL conidia suspension was dripped, 2 μL distilled water was inoculated on the injured coleoptile as control (Li et al., 2019). The incidence of wheat coleoptile was investigated after being cultured at 25°C for 10 days. For the pathogenicity experiment of tomato, the fresh plate of 5 mm was placed on tomato surface which had been wounded, and the size of disease spot on the tomato surface was observed after 3 days.

For the penetration test, the sterilized double-layer cellophane was placed on PDA, CM, MM plates, and the MM plates containing 40 μg/mL methionine or cysteine, respectively, then the fresh 5 mm disk was placed on double-layer cellophane. After being cultured at 25°C for 2 days, the cellophane on the medium plate was removed and then cultured at 25°C for 12-24 hours to observe whether the colony could penetrate. The experiment was repeated three times.

### DON content and Tri5, Tri6 gene expression assay

In order to determine DON content of strains, the conidia solution of PH-1 and *FgMet3*, *FgMet14* deletion mutants (2×10^3^ conidia/mL) were transferred into the toxin production medium GYEP and cultured at 28°C, 175rpm in the dark condition for 7 days (Bian et al., 2021). Then the DON content of strains was determined by DON ELISA Plate Kit (Weisai) (Duan et al., 2020). The DON content (μg/g) of the strain was expressed by the DON content per unit weight of hyphae.

To determine the expression of Tri5, Tri6, which related to DON synthesis, the conidia of PH-1 and *FgMet3*, *FgMet14* deletion mutants were placed in GYEP medium at 28°C, 175rpm for 36 hours in the dark. The hyphae were collected, RNA was extracted by RNA extraction kit, and inversely converted to cDNA with HiScript II Q RT SuperMix (+ gDNA wiper) (Vazyme Biotech Co. Ltd)., then the expression of Tri5, Tri6 in the strain was determined by qPCR (Bian et al., 2021). FgGadph was used as the reference gene. The experiment was repeated three times.

### Western blot assay and Co- IP assay

After the strain was shaken in YEPD medium for 36 hours, the fresh mycelium was collected, and the total protein was extracted. The extraction method was as described before (Gu et al., 2015; Yun et al., 2015). The resulting proteins were separated by 10% SDS polyacrylamide gel electrophoresis (SDS-PAGE) and transferred onto a polyvinylidene fluoride membrane with a Bio-Rad electroblotting apparatus (Tang et al., 2020).

For co-immunoprecipitation assay, the FgMet3-3×FLAG vector was transformed into the protoplasts of PH-1 and FgMet14-GFP, respectively, then FgMet3-3×FLAG strain and FgMet14-GFP+FgMet3-3×FLAG double-labeled strain were obtained. All the strains were verified by western blot assay. The total proteins of all strains were extracted and incubated with magnetic beads added with GFP antibodies. The proteins eluted from agarose were detected by Western blotting with polyclonal antibody anti-Flag or anti-GFP, and all protein samples were detected with anti-GADPH antibody as a reference, then incubated with the second antibody and detected by chemiluminescence. The experiment was repeated three times.

### Yeast two-hybrid assays

Yeast two-hybrid assays (Y2H) were performed as previously described (Wu et al., 2022). The coding sequence of each tested gene was amplified from the cDNA of PH-1 and inserted into the yeast GAL4-binding domain vector pGBKT7 and GAL4 activation domain vector pGADT7 (Clontech). The pairs of plasmids were cotransformed into *S. cerevisiae* AH109 following the LiAc/SS-DNA/PEG transformation protocol. The plasmid pairs pGBKT7-Lam and pGADT7 served as a negative control and Plasmid pairs pGBKT7-53 and pGADT7 were used as a positive control. Transformants were grown on synthetic dropout medium (SD) lacking Leu and Trp at 30°C for 3 days, and then transferred to SD without His, Leu, Trp and Ade. Other vectors were obtained using similar methods. This experiment was performed three times.

### Microscopic examinations

The fresh plates of FgMet3-GFP and FgMet14-GFP strains were cultured in YEPD medium for 12-24 h and MBL medium for 3 d, respectively. Then, the localization of FgMet3 and FgMet14 in mycelial growth and conidial stage was observed under Leica TCS SP8 confocal microscope.

### Data analysis

All of the data were analyzed using DPS 7.05 (Data Processing System) software in this research. Fisher’s least significant difference (LSD) test (P = 0.05) was used to analyze significant difference among different treatments. Fieller’s theorem was used to obtain the standard deviation of the data.

## Results

### Identification of FgMet3, FgMet14

We found FgMet3 (fgsg_08875) and FgMet14 (fgsg_01329) proteins in *Fusarium graminearum*, which are homologous to sulfate adenylyltransferase (Met3) and adenylyl-sulfate kinase (Met14) in *Saccharomyces cerevisiae* by blast (Masselot and De Robichon-Szulmajster, 1975). *FgMet3* and *FgMet14* encode 574 and 207 amino acids, respectively, and share 58.5% and 68.69% homology with Met3 and Met14 in *S. cerevisiae*. Phylogenetic tree analysis showed that both FgMet3 and FgMet14 had high homology with other *Fusarium* groups and were divided into the same group but had low homology with other fungi (**Figure S2**). Both FgMet3 and FgMet14 contain an APS_kinase domain, while FgMet3 also contains a PUA_2 domain and an ATP_sulfurylase domain, which is similar to other fungi (**Figure S2**).

### The interaction between FgMet3 and FgMet14

**Figure 1:**
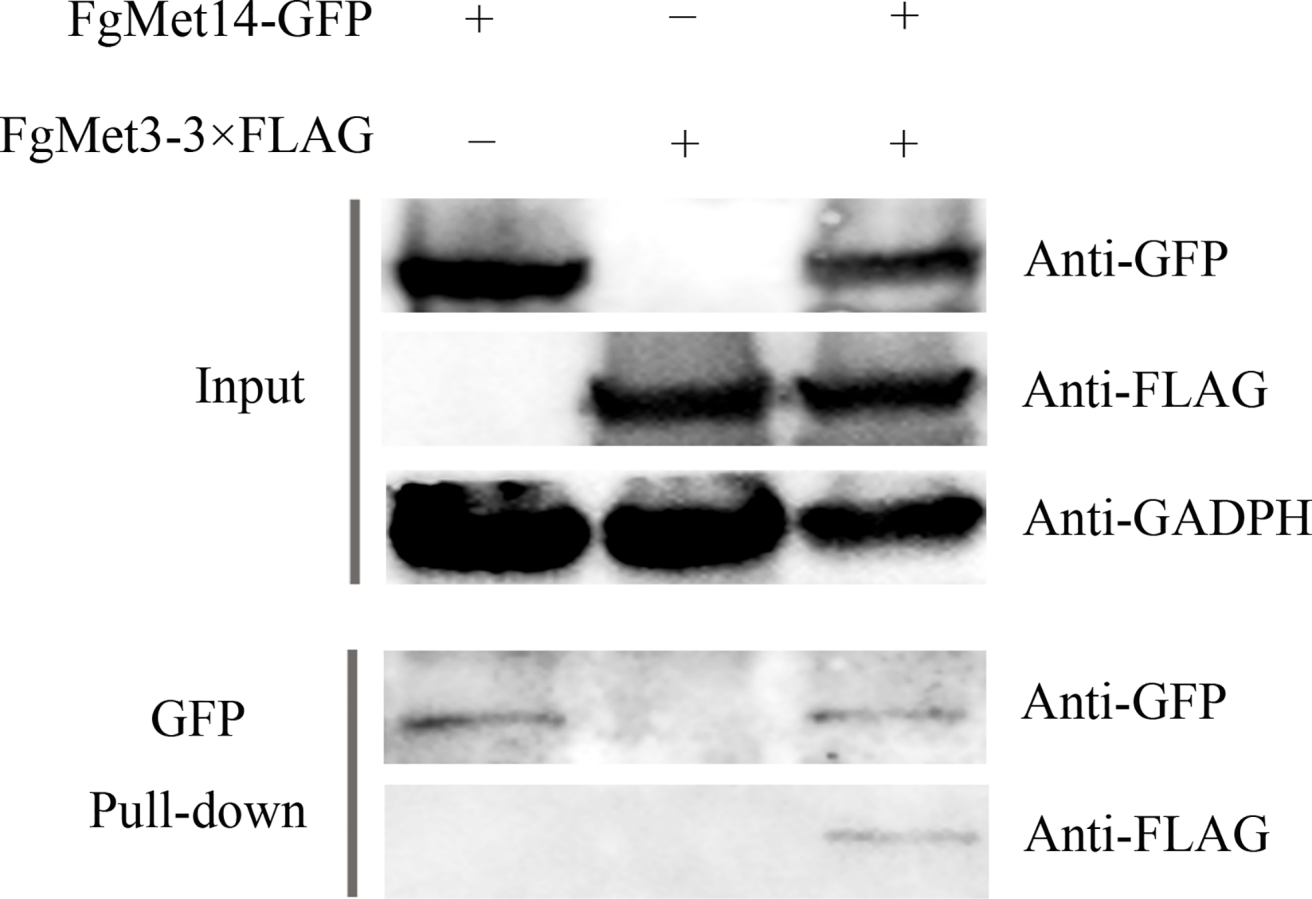
FgMet3 interacts with FgMet14. Co-immunoprecipitation (Co-IP) assay confirmed the interaction between FgMet3 and FgMet14.

To test whether FgMet3 and FgMet14 interact with each other in *F. graminearum*, a Co-IP assay and a yeast two-hybrid assay were performed. The interaction between FgMet3 and FgMet14 was further confirmed by Co-IP assay (**Figure 1**). While Yeast two-hybrid assays indicated that there may be another protein linking FgMet3 and FgMet14 (**Figure S3**).

### FgMet3 and FgMet14 are very important for vegetative growth while not necessary for conidiation of *Fusarium graminearum*

**Figure 2:**
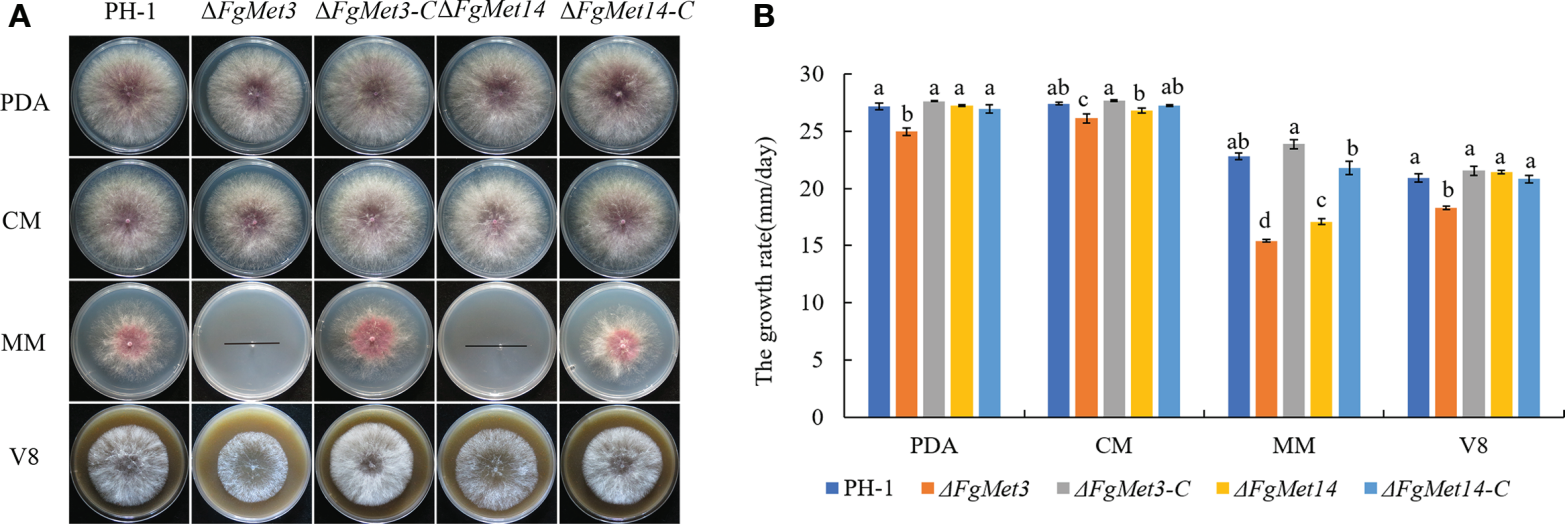
Vegetative growth of PH-1, Δ*FgMet3*, and Δ*FgMet14* mutants and their complements on different mediums. **(A)** The colonial morphology of strains on PDA, CM, MM, V8 medium at 25°C for 3 days. **(B)** Hyphal growth rate of the strains on PDA, CM, MM, V8 medium. Error bars in each column denote standard error of three repetitions. Within each treatment, bars with the same letter indicate no significant difference according to the least significant difference (LSD) test at p = 0.05.

The vegetative growth rate of the Δ*FgMet3* significantly decreased on PDA, CM, V8, and MM medium, and the vegetative growth rate of the Δ*FgMet14* significantly decreased on MM medium. Especially on MM medium, the mycelium growth rate of Δ*FgMet3* and Δ*FgMet14* became slower compared with PH-1, and the hyphae became extremely sparse, with almost no aerial hyphae, the complements Δ*FgMet3*-C and Δ*FgMet14*-C could recover the vegetative growth defects of the deleted mutants (**Figure 2**). While the conidiation of the Δ*FgMet3* and Δ*FgMet14* did not change significantly compared with PH-1 (**Table 1**).

**Table 1:** Sporulation quantity and conidia germination of PH-1, FgMet3 or FgMet14 deletion mutants and their complements.

Strain	Sporulation quantity (1×10^5^ conidia/mL)	Conidia germination rate (4 h) (%)	Conidia germination rate (8 h) (%)
PH-1	8.3 ± 0.2a	50.7 ± 1.3a	87.5 ± 1.9ab
Δ*FgMet3*	6.8 ± 0.4a	48.2 ± 1.4a	88.5 ± 1.2a
Δ*FgMet3*-C	7.1 ± 0.2a	49.0 ± 1.3a	82.3 ± 1.1b
Δ*FgMet14*	7.2 ± 0.3a	47.1 ± 2.6a	86.6 ± 2.8ab
Δ*FgMet14-*C	7.2 ± 0.1a	50.2 ± 0.7a	85.2 ± 2.1ab

Values are means and standard errors of three replicates. The same letter indicate no significant difference according to the least significant difference (LSD) test at p = 0.05.

### FgMet3 and FgMet14 are very important for the synthesis of methionine and cysteine

**Figure 3:**
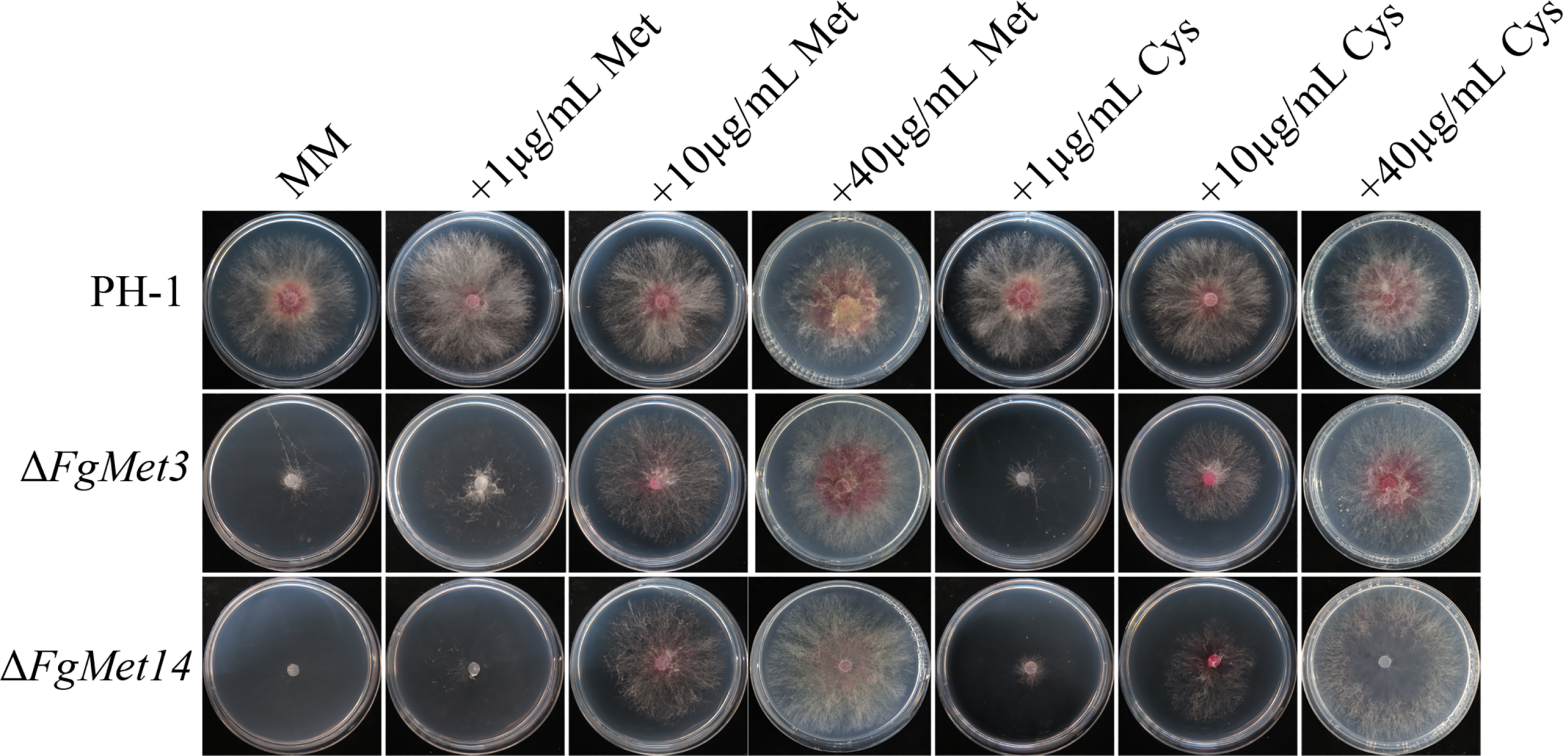
Colony diameter and morphology of PH-1, Δ*FgMet3* and Δ*FgMet14* mutants and their complements cultured on MM medium supplemented with different concentrations (1μg/mL, 10μg/mL and 40μg/mL) of cysteine and methionine. The strains were cultured on the medium at 25°C for 3 days.

We already know that met3 and met14 are very important for the synthesis of methionine and cysteine in *S. cerevisiae*. In order to explore the role of met3 and met14 in the synthesis of methionine and cysteine in *F. graminearum*, methionine and cysteine were added to MM medium. The mycelium growth rate and density of Δ*FgMet3* and Δ*FgMet14* could be partially restored, and the growth rate and density of mutant hyphae could be restored more with the increase concentration of methionine and cysteine (**Figure 3**).

### FgMet3 and FgMet14 regulate the sensitivity to different fungicides

**Figure 4:**
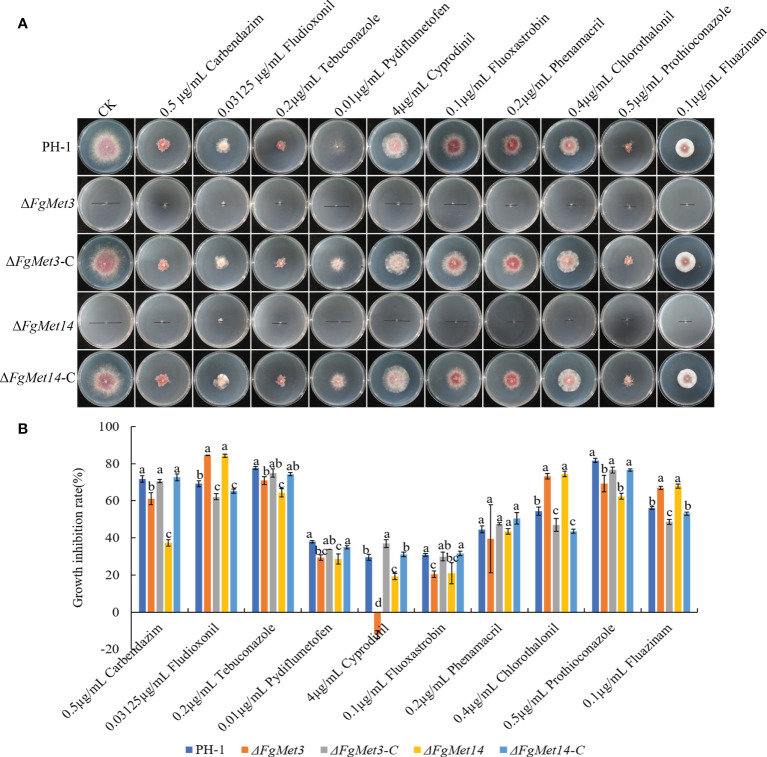
The sensitivity of Δ*FgMet3* and Δ*FgMet14* to different fungicides compared with PH-1 and complements. **(A)** Effects of 0.5 μg/mL carbendazim, 0.03125 μg/mL fludioxonil, 0.2 μg/mL tebuconazole, 0.01 μg/mL pydiflumetofen, 4 μg/mL cyprodinil, 0.1 μg/mL fluoxastrobin, 0.2 μg/mL phenamacril, 0.4 μg/mL chlorothalonil, 0.5 μg/mL prothioconazole and 0.1 μg/mL fluazinam on mycelial linear growth of Δ*FgMet3* and Δ*FgMet14* mutants. **(B)** The inhibition of the mycelial growth rate was examined after each strain was incubated for 3 days on PDA supplemented with each fungicide. Bars denote standard deviations from three experiments. Within each treatment, the same letter indicate no significant difference according to the least significant difference (LSD) test. P = 0.05.

The sensitivities of *FgMet3* and *FgMet14* deletion mutants to 0.5 μg/mL carbendazim, 0.03125 μg/mL fludioxonil, 0.2 μg/mL tebuconazole, 0.01 μg/mL pydiflumetofen, 4 μg/mL cyprodinil, 0.1 μg/mL fluoxastrobin, 0.2 μg/mL phenamacril, 0.4 μg/mL chlorothalonil, 0.5 μg/mL prothioconazole, 0.1 μg/mL fluazinam were determined (**Figure 4**). Compared with PH-1 and complements, the sensitivity of FgMet3 and FgMet14 deletion mutants to fludioxonil, chlorothalonil and fluazinam increased, while the sensitivity to carbendazim, tebuconazole, pydiflumetofen, cyprodinil, fluoxastrobin, and prothioconazole decreased, the sensitivity to phenamacril did not change significantly (**Figure 4B**). This result showed that FgMet3 and FgMet14 nonspecifically regulated the sensitivity of *F. graminearum* to different fungicides.

### FgMet3 and FgMet14 regulate the response of *Fusarium graminearum* to different stress factors

**Figure 5:**
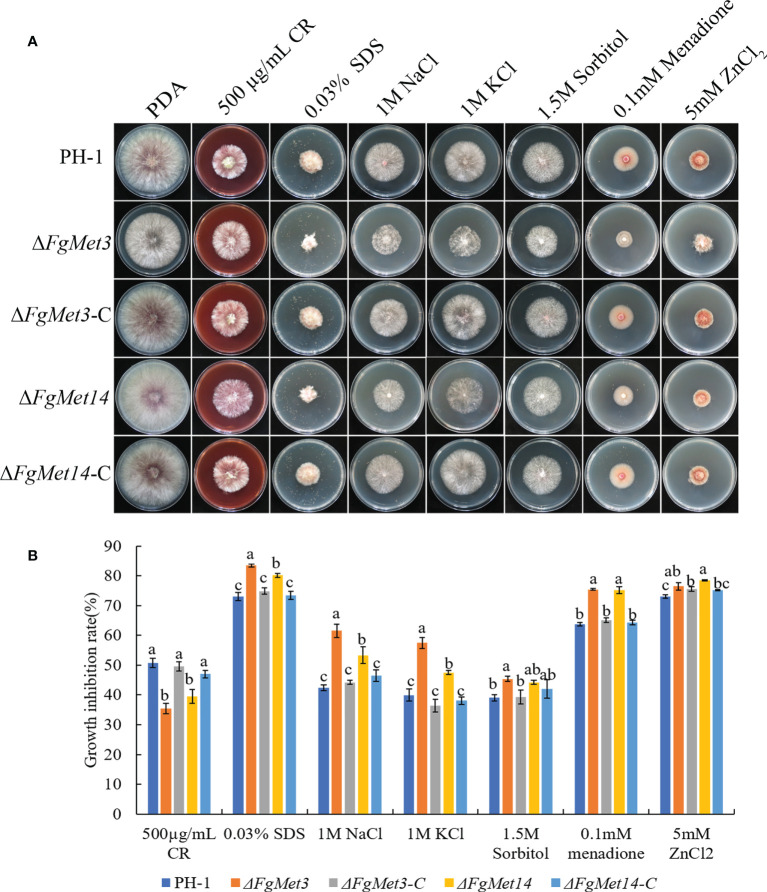
The sensitivity of Δ*FgMet3* and Δ*FgMet14* to different stress agents compared with PH-1 and complements. **(A)** The sensitivity of *Fusarium graminearum* wild-type PH-1, knockout mutants, and complementation strains to 500 µg/mL Congo red, 0.03% SDS, 1 M NaCl, 1 M KCl, 1.5 M Sorbitol, 5 mM ZnCl_2_, and 0.1 mM Menadione on PDA plate. **(B)** The inhibition of the mycelial growth rate was examined after each strain was incubated for 3 days on PDA supplemented with each stress compound. Bars denote standard deviations from three experiments. Within each treatment, bars with the same letter indicate no significant difference according to the least significant difference (LSD) test. P = 0.05.

In order to explore the sensitivity of *FgMet3* and *FgMet14* deletion mutants to different stress factors, we measured the sensitivity of FgMet3 and FgMet14 deletion mutants to cell wall stress factor 500 μg/mL Congo red, cell membrane stress factor 0.03% SDS, osmotic stress factors 1M NaCl, 1M KCl and 1.5M Sorbitol, oxidative stress factor 0.1 mM Menadione and ion stress factor 5mM ZnCl_2_ (**Figure 5**). The results showed that FgMet3 and FgMet14 deletion mutants were less sensitive to CR stress and more sensitive to SDS stress, NaCl stress, KCl stress, Sorbitol stress, Menadione, and Zn ion stress compared with PH-1 (**Figure 5**). It can be seen that FgMet3 and FgMet14 regulate the response of *F. graminearum* to different stress factors.

### FgMet3 and FgMet14 are necessary for pigment formation in *Fusarium graminearum*

**Figure 6:**
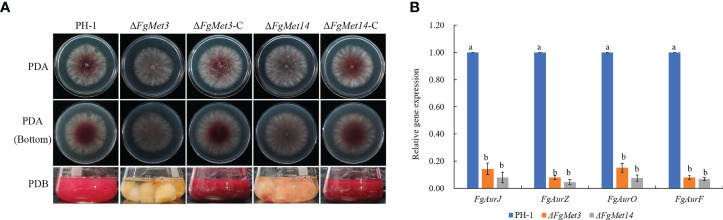
Pigment formation of PH-1, FgMet3 or FgMet14 deletion mutants and their complements. **(A)** All the strains were cultured on PDA medium for 2d and cultured in PDB medium for 3d to produce pigment. **(B)** Relative expression levels of Aurofusarin biosynthesis-related genes FgAurJ, FgAurZ, FgAurF, and FgAurO in the Δ*FgMet3* and Δ*FgMet14* mutants compared with the WT strain. Bars with the same letter indicate no significant difference according to the least significant difference (LSD) test. P = 0.05.

After culturing fresh fungus plates on PDA medium for 2 days, we found that *FgMet3* and *FgMet14* deletion mutants produced less red pigment than PH-1 and complements. After fresh fungus plates were cultured in PDB medium for 3 days, *FgMet3* and *FgMet14* deletion mutants also produced less red pigment than PH-1, while replicates could restore the level of red pigment production (**Figure 6A**). In order to prove this result, we determined expression levels of four aurofusarin biosynthesis-related genes *AurJ*, *AurZ*, *AurF*, and *AurO* (Frandsen et al., 2011; Ding et al., 2018). QRT-PCR assays confirmed that these genes were down-regulated significantly in Δ*FgMet3* and Δ*FgMet14* (**Figure 6B**).

### FgMet3 and FgMet14 regulate the sexual reproduction of *Fusarium graminearum*

**Figure 7:**
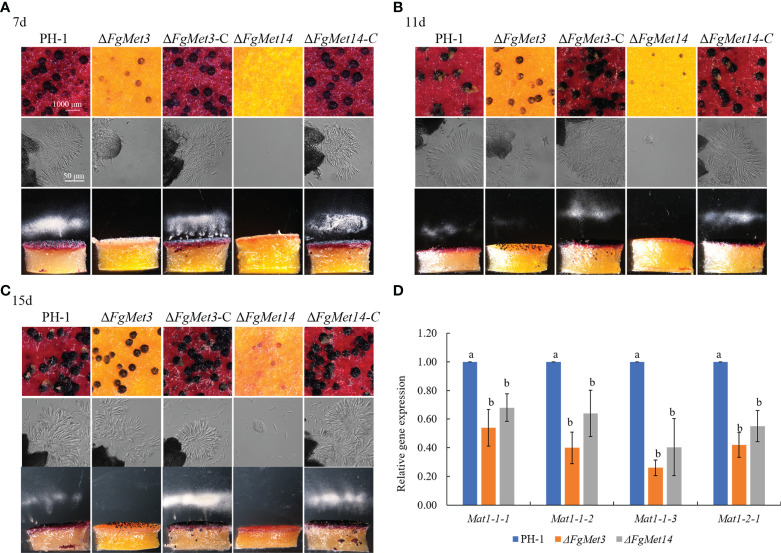
Sexual reproduction of PH-1, FgMet3 or FgMet14 deletion mutants and their complements. **(A)** The sexual reproduction of strains when incubated on carrot medium for 7 days. **(B)** The sexual reproduction of strains when incubated on carrot medium for 11 days. **(C)** The sexual reproduction of strains when incubated on carrot medium for 15 days. **(D)** Relative expression levels of sexual reproduction-related genes Mat1-1-1, Mat1-1-2, Mat1-1-3, and Mat1-2-1 in the Δ*FgMet3* and Δ*FgMet14* mutants compared with the WT strain. Bars with the same letter indicate no significant difference according to the least significant difference (LSD) test. P = 0.05.

In order to explore the role of FgMet3 and FgMet14 in the sexual reproduction of *F. graminearum*, we measured the sexual reproductive ability of FgMet3 and FgMet14. The results showed that the development of perithecium was slower and matured later in *FgMet3* and *FgMet14* deletion mutants than in PH-1. After being cultured on carrot medium (Tang et al., 2020) for 7 days, *FgMet3* deletion mutant could produce smaller immature perithecium, but could not produce and release ascospores. while *FgMet14* deletion mutant could not produce perithecium on the 7th day (**Figure 7A**). When cultured on carrot medium for 11 days, the perithecium of *FgMet3* deletion mutant was still immature, could produce and release a small number of ascospores but could not form asci composed of 8 ascospores. While *FgMet14* deletion mutant could produce very small perithecium but could not produce and release ascospores (**Figure 7B**). When cultured on carrot medium for 15 days, *FgMet3* deletion mutant could produce mature perithecium and asci composed of 8 ascospores. The ascospores were forced to release in the shape of petals, but the FgMet3 deletion mutant could not actively release ascospores. *FgMet14* deletion mutants could not produce mature ascospores and could not produce and release ascospores on the 15th day (**Figure 7C**). Because that MAT1-1-1, MAT1-1-2, MAT1-1-3, and MAT1-2-1 were essential for sexual reproduction in *F. graminearum* (Kim et al., 2012), we determined the four gene expressions in PH-1 and deletion mutants. Results showed that these genes were down-regulated significantly in Δ*FgMet3* and Δ*FgMet14* (**Figure 7D**).

**Figure 8:**
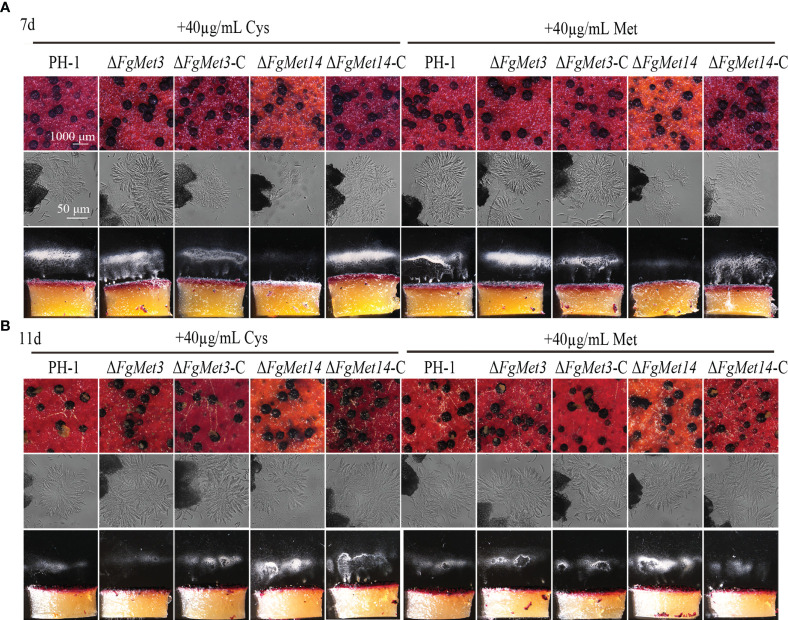
Exogenous addition of cysteine and methionine could partially restore the sexual reproductive ability of FgMet3 and FgMet14 deletion mutants. **(A)** The sexual reproduction of strains when incubated on carrot medium which contains 40µg/mL cysteine or methionine for 7 days. **(B)** The sexual reproduction of strains when incubated on carrot medium which contains 40µg/mL cysteine or methionine for 11 days.

After exogenous addition of 40 μg/mL cysteine or methionine, *FgMet3* and *FgMet14* deletion mutants could produce and release ascospores after 7 days on carrot medium (**Figure 8A**). After 11 days of culture on carrot medium, *FgMet3* and *FgMet14* deletion mutants could produce mature perithecium, form asci composed of 8 ascospores and release ascospores compared with PH-1 (**Figure 8B**). Therefore, exogenous addition of cysteine and methionine can partially restore the developmental process and maturity of *FgMet3* and *FgMet14* deletion mutants. The results showed that FgMet3 and FgMet14 played a crucial role in the sexual reproduction of *F. graminearum*.

### FgMet3 and FgMet14 are important for the pathogenicity and penetration while not crucial for DON biosynthesis of *Fusarium graminearum*

**Figure 9:**
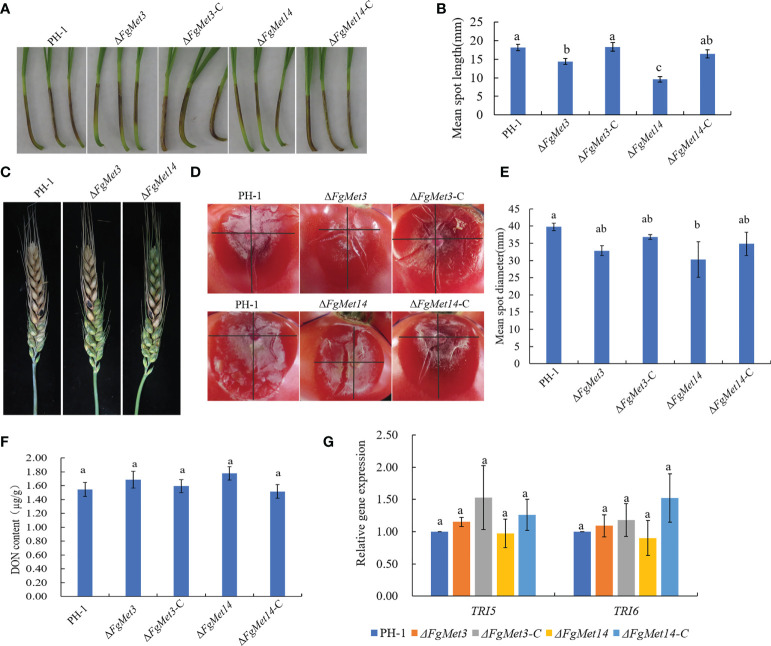
Defects of Δ*FgMet3* and Δ*FgMet14* mutants in plant infection. **(A)** Wheat coleoptiles were examined at 10 days after inoculated with a conidial suspension. **(B)** Mean spot length of the strains. Bars with the same letter indicate no significant difference at p = 0.05. **(C)** Flowering wheat heads were examined at 14 days post-inoculation (dpi). The inoculation sites were indicated by black dots. **(D, E)** The tomato mean spot diameter were examined at 3 days after placed with fresh dishes. **(F)** FgMet3 and FgMet14 are not important for DON biosynthesis of *Fusarium graminearum*. All strains were incubated in GYEP for 7 days and mycelia were harvested and dry for weighing. **(G)** FgMet3 and FgMet14 didn’t regulate TRI5, TRI6 gene expression in *Fusarium graminearum*. All strains were incubated in GYEP for 36 h and mycelia were harvested for qRT-PCR. Bars with the same letter indicate no significant difference according to the least significant difference (LSD) test at p = 0.05.

We determined the pathogenicity of *FgMet3* and *FgMet14* deletion mutants to wheat head and wheat coleoptile. In the wheat coleoptile pathogenicity experiment, 10 days after inoculation on injured wheat coleoptile, the lesion length of wheat coleoptile infected by *FgMet3* and *FgMet14* deletion mutants was significantly lower than that of PH-1 (**Figures 9A, B**). In the wheat head pathogenicity experiment, 14 days after wheat head inoculation, the results showed that the pathogenicity of *FgMet14* deletion mutants decreased compared with PH-1, while there was no significant change in pathogenicity of *FgMet3* deletion mutants (**Figure 9C**). In the tomato pathogenicity experiment, fresh fungal plates were inoculated on the injured tomato surface for 3 days. The results showed that the pathogenicity of *FgMet14* deletion mutant decreased significantly compared with PH-1, while the pathogenicity of *FgMet3* deletion mutant did not change significantly (**Figures 9D, E**). The results of the pathogenicity test showed that FgMet3 and FgMet14 were important to the pathogenicity of *F. graminearum*.

Considering that DON is an important virulence factor of *F. graminearum*, we determined the DON content of *FgMet3* and *FgMet14* deletion mutants in this study. The results showed that there was no significant change in DON content of Δ*FgMet3* and Δ*FgMet14* mutants compared with PH-1. At the same time, we also measured the gene expression of TRI5, TRI6, which related to DON synthesis, and found no significant change in TRI5, TRI6 gene expression between Δ*FgMet3* and Δ*FgMet14* mutants and PH-1. These results suggested that FgMet3 and FgMet14 did not play an important role in DON biosynthesis and TRI5, TRI6 gene expression in *F. graminearum* (**Figures 9F, G**).

**Figure 10:**
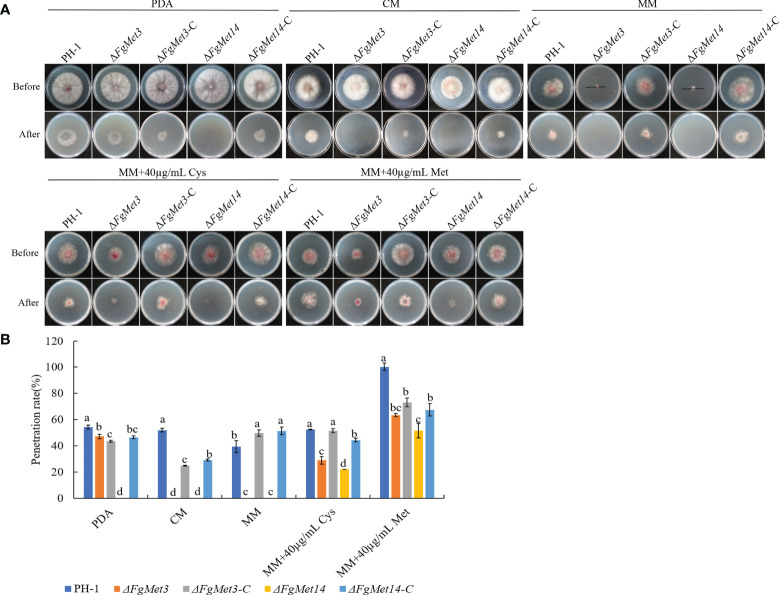
Defects of Δ*FgMet3* and Δ*FgMet14* mutants in plant penetration. **(A)** The penetration of strains. The sterilized double-layer cellophane was placed on PDA, CM, MM plates, and the MM plates containing 40 µg/mL methionine or cysteine for this experiment. **(B)** The penetration rate of strains. The penetration rate (%) = mean diameter after penetration/mean diameter before penetration × 100. Bars with the same letter indicate no significant difference according to the least significant difference (LSD) test at p = 0.05.

Because the deletion of *FgMet3* and *FgMet14* had no significant effect on the spore production and conidia germination of *F. graminearum*, we speculated that the decrease of pathogenicity of Δ*FgMet3* and Δ*FgMet14* was due to the decrease of penetration. In order to confirm this hypothesis, we measured the penetration of Δ*FgMet3* and Δ*FgMet14* mutants. After removing the cellophane for 24-48 hours, the results showed that the *FgMet3* deletion mutant could penetrate as well as PH-1 on PDA medium, while Δ*FgMet14* mutant couldn’t penetrate. On CM and MM medium, *FgMet3* and *FgMet14* deletion mutants could not penetrate. When 40 μg/mL cysteine or methionine was added to MM medium, *FgMet3* and *FgMet14* deletion mutants could restore the penetration ability, and the penetration ability of Δ*FgMet3* mutants recovered more. This result revealed the importance of FgMet3 and FgMet14 for the penetration of *F. graminearum* (**Figure 10**).

### Subcellular localization of FgMet3 and FgMet14

**Figure 11:**
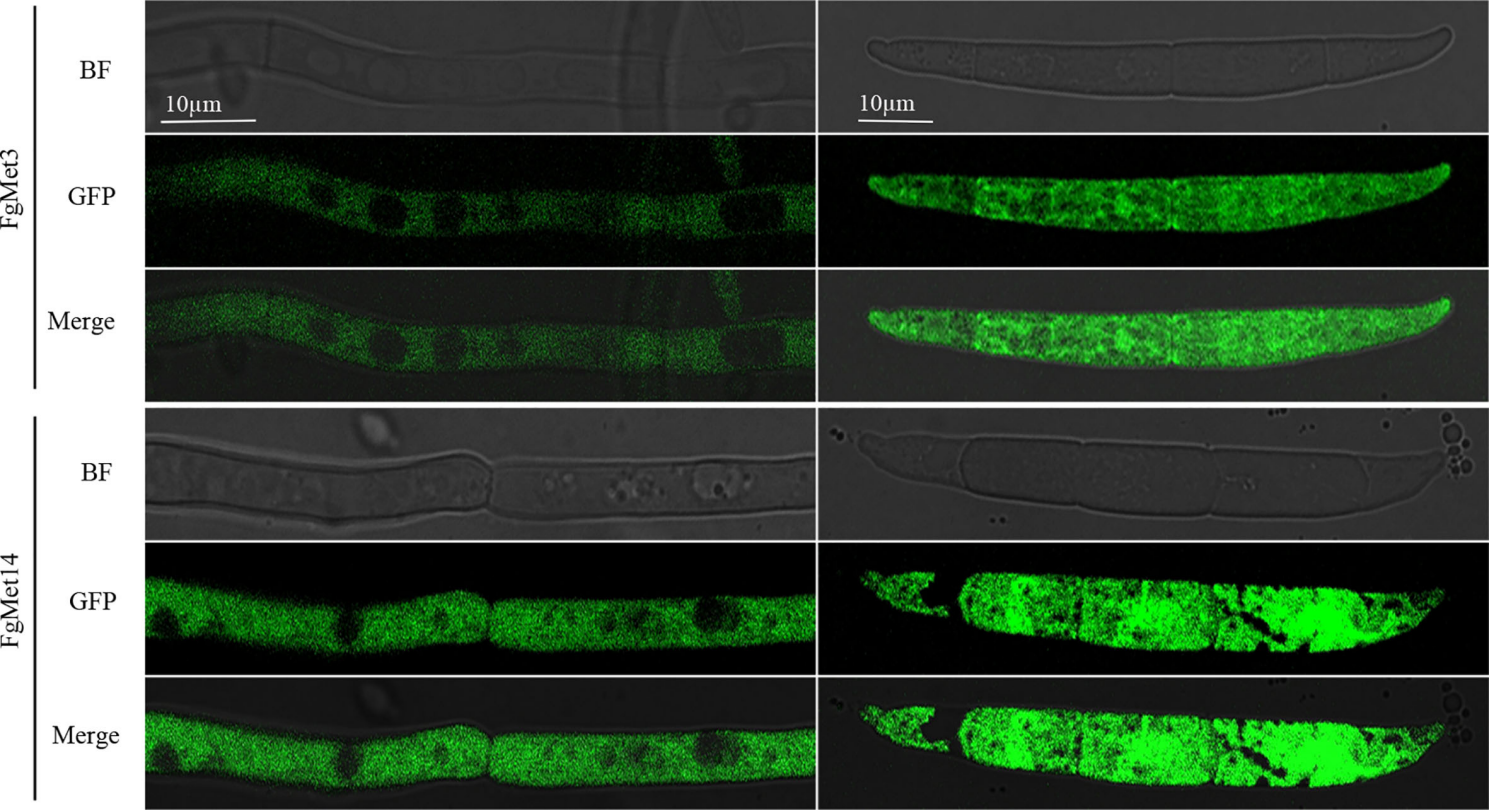
Subcellular localization of FgMet3 and FgMet14. FgMet3 and FgMet14 were localized to the cytoplasm. All strains were incubated in YEPD for 12-24 h and MBL for 3 d to obtain mycelium and conidia.

We constructed FgMet3-GFP and FgMet14-GFP strains and observed their localization in the vegetative mycelia growth and conidia stage. It was found that FgMet3 and FgMet14 were located in the cytoplasm in both vegetative mycelia growth and conidia stage (**Figure 11**).

## Discussion

*Fusarium graminearum* can cause wheat scab and other fungal diseases on the main grain crops in the world. In addition, *F. graminearum* can produce mycotoxins, reduce grain yield and quality, and threaten food safety (Luo et al., 2021). Because of the severe resistance problem caused by traditional fungicides, some new and high-efficient fungicides need to be developed (Becher et al., 2010). Understanding the chemical components of the signaling networks involved in fungal growth and infection could provide potential targets for developing novel compounds. In this study, we identified sulfate adenylyltransferase (FgMet3) and adenylyl-sulfate kinase (FgMet14) in *F. graminearum* and explored their biological functions.

In *S. cerevisiae*, *FgMet3* encodes ATP sulfurylase, which catalyzes the initial steps of the sulfur assimilation pathway (Mendoza-Cozatl et al., 2005). The sulfur assimilation pathway leads to the formation of hydrogen sulfide in the biosynthesis of homocysteine, cysteine, and methionine (Ullrich et al., 2001). In *Aspergillus nidulans* and *Neurospora crassa*, the inorganic sulfate is first phosphorylated by adenosine triphosphate (ATP) in two enzymatic steps to form PAPS, which is reduced to sulfite and then condensed with O-acetylserine to form cysteine, which is also an intermediate for the synthesis of methionine and S-adenosylmethionine (Marzluf, 1997; Paietta, 2016). Cysteine and methionine play an essential role in protein synthesis and cell life activities (Thomas and SurdinKerjan, 1997).

Previous studies have shown that cysteine deficiency mutants can only grow when cysteine is present in the culture medium, and methionine synthase is essential for *Aspergillus fumigatus* growth (Amich, 2022). In our study, the vegetative growth rate of the Δ*FgMet3* significantly decreased on PDA, CM, V8, and MM medium, and the vegetative growth rate of the Δ*FgMet14* significantly decreased on MM medium (**Figure 2**). Especially on MM medium, Δ*FgMet3* and Δ*FgMet14* showed significant growth defects compared with wild-type strains. The vegetative growth defect of Δ*FgMet3* and Δ*FgMet14* on MM medium is due to the lack of important nutrients, including cysteine and methionine. On the nutrient-rich medium, Δ*FgMet3* and Δ*FgMet14* can absorb cysteine and methionine needed for their growth, thus partially restoring the growth rate. The growth defects of the mutants were partially repaired by adding exogenous cysteine and methionine to MM medium, and the repair degree of growth defects increased with the increase of exogenous concentration (**Figure 3**), which indicated the importance of FgMet3 and FgMet14 in cysteine and methionine biosynthesis.

The sexual cycle plays a dominant role in the growth and development of *F. graminearum*, and its sexual offspring (ascospores) are important overwintering and transmitting substances to complete the disease cycle (Cavinder et al., 2012; Son et al., 2017). Therefore, the sexual reproduction of *F. graminearum* is very important to complete the cycle of plant diseases, and nutritional supply is crucial for the sexual reproduction of *F. graminearum* (Kim et al., 2015; Kim et al., 2021). Our results showed that the deletion of *FgMet3* and *FgMet14* seriously affected the sexual reproduction of *F. graminearum* (**Figure 7**). The perithecium maturity and ability to eject ascospores were affected in Δ*FgMet3* and Δ*FgMet14* mutants. This may be due to the decreased expression of genes related to sexual reproduction in the deletion mutants. While, exogenous addition of cysteine and methionine can partially restore the developmental process and maturity of *FgMet3* and *FgMet14* deletion mutants (**Figure 8**). The results indicated that FgMet3 and FgMet14 played a crucial role in sexual reproduction of *F. graminearum*.

Because of the important role of sexual reproduction of *F. graminearum* in the infection cycle, we determined the pathogenicity of *FgMet3* and *FgMet14* deletion mutants to the wheat head and wheat coleoptile. In *A. fumigatus*, the virulence of cysteine and methionine deficiency mutants decreased (Amich, 2022). In this study, the results showed that the pathogenicity of FgMet14-deficient mutant was significantly lower than that of PH-1, and the pathogenicity of *FgMet3*-deficient mutant had no significant change After 14 days of inoculation on the head of wheat. In the pathogenicity experiment of Wheat Coleoptile, ten days after inoculation on injured wheat coleoptile, the lesion length of wheat coleoptile infected by Δ*FgMet3* and Δ*FgMet14* mutants was significantly lower than that of PH-1. In tomato pathogenicity experiment, fresh plates were inoculated on the injured tomato surface for 3 days. The results showed that the pathogenicity of Δ*FgMet14* mutant decreased significantly, while the pathogenicity of Δ*FgMet3* mutant did not change significantly compared with PH-1 (**Figure 9**).

DON is an important virulence factor, but in our study, the DON content of *FgMet3* or *FgMet14* deletion mutants did not change significantly. Therefore, the decrease of pathogenicity of Δ*FgMet3* and Δ*FgMet14* may be related to the decrease of their penetration power. The result of the penetration experiment showed that under the condition of nutrient deficiency, the *FgMet3* and *FgMet14* deletion mutants lost their penetration power. When exogenous cysteine and methionine were added to the culture medium, both *FgMet3* and *FgMet14* deletion mutants recovered their penetration power, and the penetration power of Δ*FgMet3* mutant recovered more (**Figure 10**). When cultured on nutrient-rich PDA medium, Δ*FgMet3* mutant could penetrate, while Δ*FgMet14* mutant could not. when cultured on the same nutrient-rich CM medium, Δ*FgMet3* and Δ*FgMet14* mutants could not penetrate (**Figure 10**). These results showed that under the condition of rich nutrition, Δ*FgMet3* and Δ*FgMet14* mutants would selectively absorb nutrients from the outside world, but Δ*FgMet3* mutant are easier to use external nutrition than *FgMet14* deletion mutant. Therefore, we speculated that the *FgMet3* and *FgMet14* deletion mutants could not make use of the nutrients (including cysteine and methionine) on the coleoptile of wheat, so that the pathogenicity could not be recovered, while the *FgMet3* deletion mutant could use the nutrients in wheat heads and tomatoes to restore penetration to complete infection.

In addition, FgMet3 and FgMet14 nonspecifically regulate the sensitivity of *F. graminearum* to fungicides, the sensitivity of Δ*FgMet3* and Δ*FgMet14* mutants to fludioxonil, chlorothalonil and fluazinam increased, while the sensitivity to carbendazim, tebuconazole, pydiflumetofen, cyprodinil, fluoxastrobin, and prothioconazole decreased (**Figure 4**). This may because some fungicides have the same effect on cysteine and methionine biosynthesis while others have the opposite effect. Moreover, FgMet3 and FgMet14 regulate chromogenesis and the sensitivity to different stresses in *F. graminearum*, Δ*FgMet3* and Δ*FgMet14* mutants were less sensitive to CR stress and more sensitive to SDS, NaCl, KCl, Sorbitol, Menadione, and Zn ion stress compared with PH-1 (**Figures 5**, **6**).

**Figure 12:**
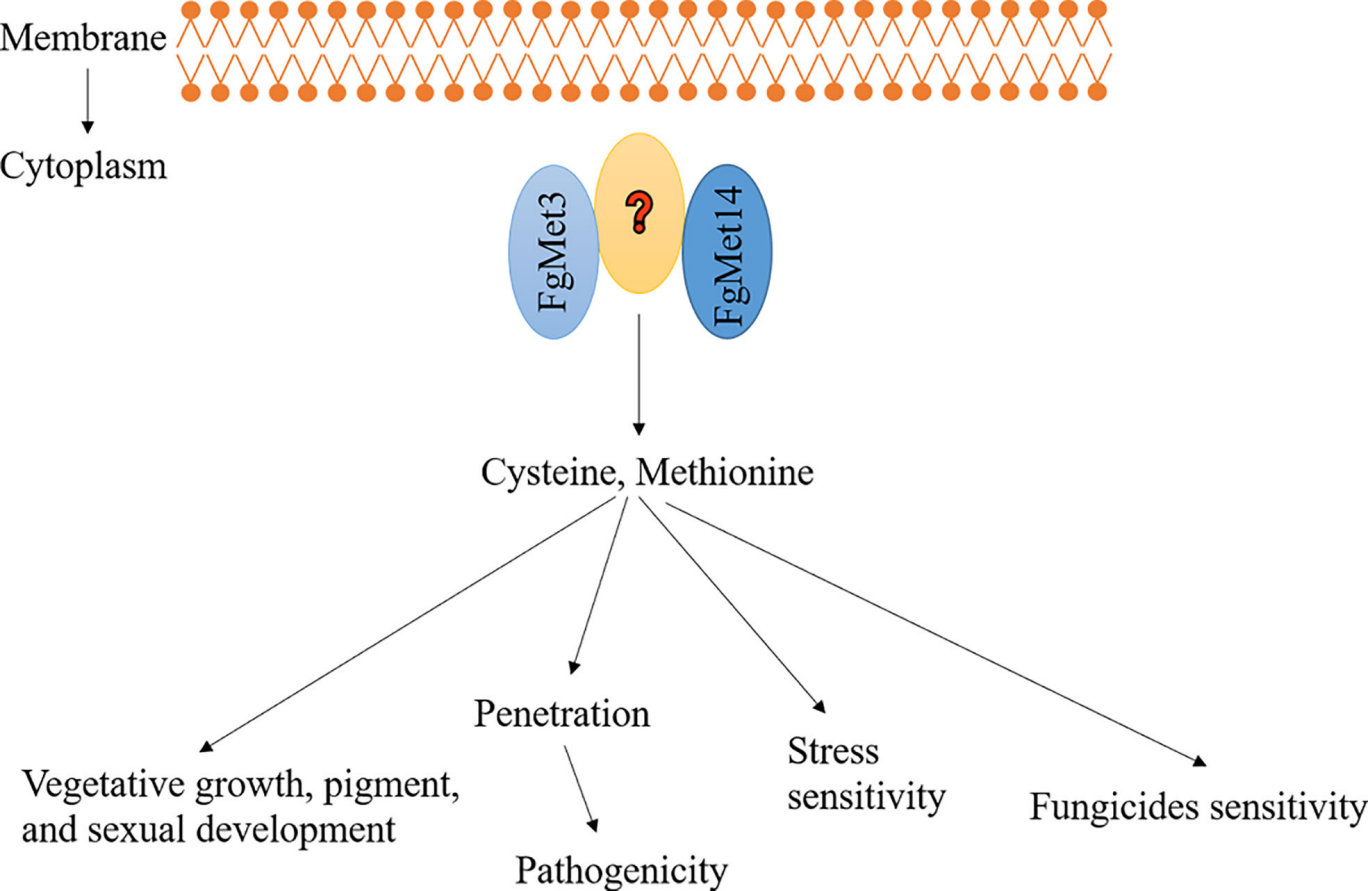
A proposed model showing the function of FgMet3 and FgMet14. FgMet3 interacts with FgMet14 indirectly, and they regulate development, pathogenicity, pigment formation, sensitivity to fungicides and stress factors of *F. graminearum*.

According to these results, we proposed a model about the function of FgMet3 and FgMet14 in *F. graminearum* (**Figure 12**): FgMet3 interacts with FgMet14 indirectly, and they participate in the sulfur assimilation pathway together, any one of them is missing, the sulfur assimilation pathway will be destroyed, affecting the synthesis of methionine and cysteine. *FgMet3* and *FgMet14* deletion mutants have certain vegetative growth and chromogenesis defects, delay sexual development, and could not normally release ascospores. In addition, the deletion of FgMet3 and FgMet14 will also affect pathogenicity, the sensitivity of *F. graminearum* to many fungicides and different stress factors. This study is helpful for us to understand the nutritional mechanism related to the development of fungi and provide new ideas for the research of new agents.

## Data availability statement

The original contributions presented in the study are included in the article/**Supplementary Material**. Further inquiries can be directed to the corresponding author.

## Author contributions

Y-PH designed the study. M-GZ gave good suggestions foranalyzing results. F-FZ did the most work about the deletion of fgmet3 and fgmet14, analyzing their functions and interaction with plants. Z-LY, W-W, Z-YH and X-WM did some word about the phenotype analysis about the deletion mutant of fgmet3 and fgmet14. All authors contributed to the article and approved the submitted version.

## Funding

This study was sponsored by National Natural Science Foundation of China (31972307), Jiangsu Agricultural Science and Technology Innovation Fund (CX (21)2037), “Six Talent Peaks” Project in Jiangsu Province (NY-040).

## Conflict of interest

The authors declare that the research was conducted in the absence of any commercial or financial relationships that could be construed as a potential conflict of interest.

## Publisher’s note

All claims expressed in this article are solely those of the authors and do not necessarily represent those of their affiliated organizations, or those of the publisher, the editors and the reviewers. Any product that may be evaluated in this article, or claim that may be made by its manufacturer, is not guaranteed or endorsed by the publisher.
