# Supplementary material for: FgMet3 and FgMet14 related to cysteine and methionine biosynthesis regulate vegetative growth, sexual reproduction, pathogenicity, and sensitivity to fungicides in Fusarium graminearum
Source: Front Plant Sci. 2022 Oct 24;13:1011709. doi: 10.3389/fpls.2022.1011709 (PMC9638117; doi:10.3389/fpls.2022.1011709)
Supplement: Supplementary file 1 [file Table_1.docx]

Supplementary Figures


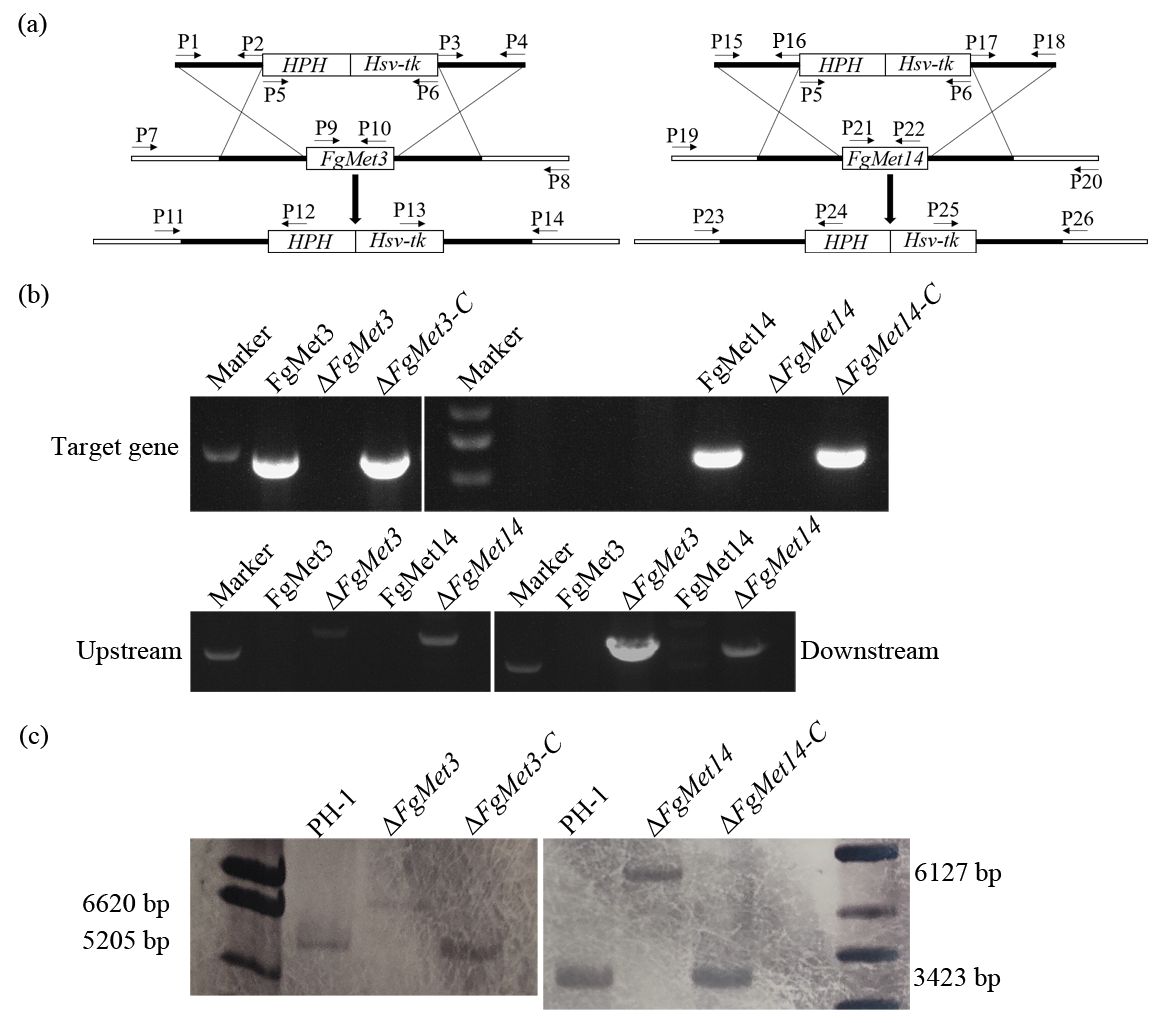


Fig.S1 Generation and confirmation of FgMet3/FgMet14 gene deletion mutant. (a) Gene deletion strategy for FgMet3 and FgMet14. Primer pairs P1/P2 and P3/P4 were used to amplify the flanking fragments about 1kb upstream and downstream of FgMet3. Primer pairs P5/P6 were used to amplify double sieve gene fragment. Primer pairs P9/P10, P11/P12, and P13/P14 were used to verify the target fragment, the insert position of upstream and downstream of FgMet3. Primer pairs P15/P16 and P17/P18 were used to amplify the flanking fragments about 1kb upstream and downstream of FgMet14. Primer pairs P21/P22, P23/P24, and P25/P26 were used to verify the target fragment, the insert position of upstream and downstream of FgMet14. (b) Confirmation of FgMet3/FgMet14 deletion mutants and their complements by PCR strategy. Δ*FgMet3* and Δ*FgMet14* were the deletion mutants, and Δ*FgMet3*-C and Δ*FgMet14*-C were the complements. (c) Southern blot analysis of PH-1, FgMet3/FgMet14 deletion mutants, and their complements. Total DNA of PH-1, Δ*FgMet3,* and Δ*FgMet3*-C was digested by EcoR I, Probe about 633 bp was located in downstream of FgMet3; Total DNA of PH-1, Δ*FgMet14,* and Δ*FgMet14*-C was digested by Hind III, Probe about 610 bp was located in downstream of FgMet14.


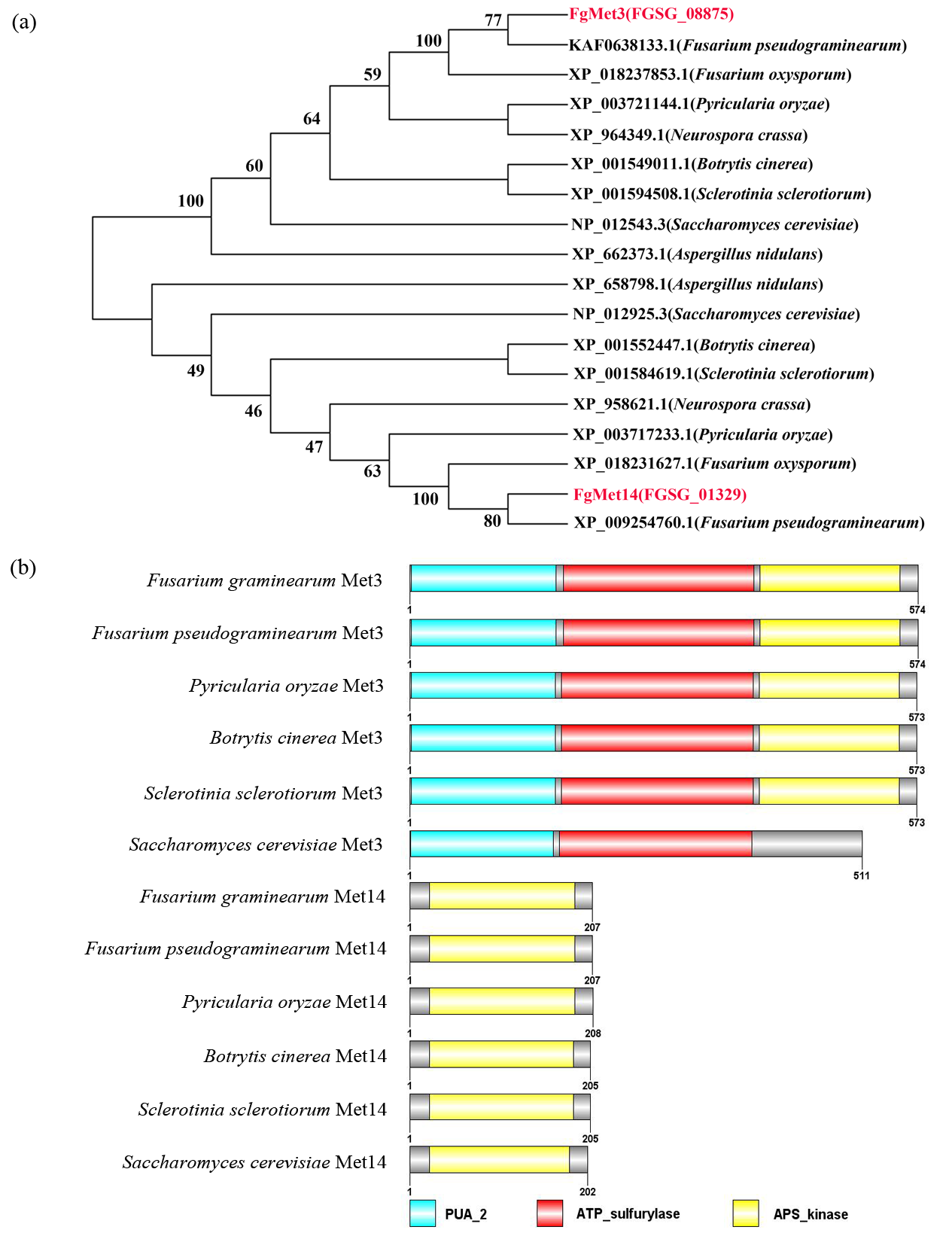


Fig.S2 Identification of the FgMet3 and FgMet14 in *Fusarium graminearum.* (a) Phylogenetic analysis of Met3 and Met14 in different fungi. Amino acid sequences were aligned by BLAST and a neighbor-joining tree was generated using MEGA 7. (b) The domain of Met3 and Met14 in *Fusarium graminearum* and other fungi. Simple modular architecture research tool (SMART) database was used to find the domain of Met3 and Met14.


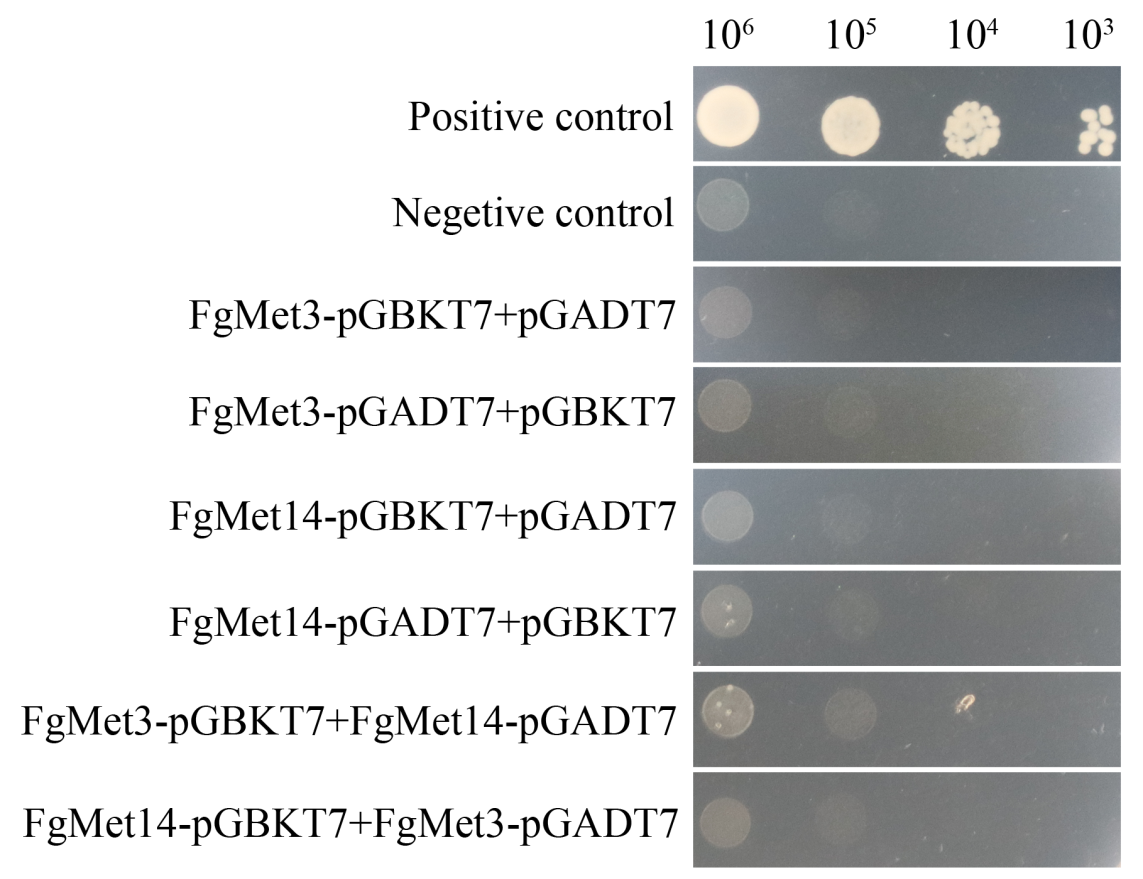


Fig.S3 Yeast two-hybrid analysis of the interaction between FgMet3 and FgMet14. Different concentrations of yeast transformants were assayed for growth on SD−Trp−Leu−His-Ade plates.
