# Supplementary material for: FgMet3 and FgMet14 related to cysteine and methionine biosynthesis regulate vegetative growth, sexual reproduction, pathogenicity, and sensitivity to fungicides in Fusarium graminearum
Source: Front Plant Sci. 2022 Oct 24;13:1011709. doi: 10.3389/fpls.2022.1011709 (PMC9638117; doi:10.3389/fpls.2022.1011709)
Supplement: Supplementary file 2 [file Table_2.doc]

Supplementary Table

***Table S1*** *PCR primers used in the construction of* *ΔFgMet3 and ΔFgMet14*

| **Primer** | **Sequence (5’-3’)** | **Relevant characteristics** |
| --- | --- | --- |
| P1 | GAAGAATGGATGCTTGTGC | PCR primers to amplify FgMet3 upstream fragment |
| P2 | CCTTCAATATCATCTTCTGTTG  TGTTGTGAGGGTTTCTG |
| P3 | GGAGACAATACCGGAAGGAA  GCGATGATTTTTGACGAC | PCR primers to amplify FgMet3 downstream fragment |
| P4 | ATTCCTCACCATCATTCGAG |
| P5 | ACAGAAGATGATATTGAAGG | PCR primers to amplify HPH-Hsv-tk fragment |
| P6 | TTCCTTCCGGTATTGTCTCC |
| P7 | GATGGATACGGAGATGCAGA | PCR primers to amplify FgMet3 deletion fragment |
| P8 | CTCGTTTATGTCTGACTTCC |
| P9 | TCCTCATGGTGGTGTCCTA | PCR primers to verify the target fragment of FgMet3 |
| P10 | GTGAAGTGGTCGATGTCAC |
| P11 | TGGTCAGCATCTGACCACCT | PCR primers to verify the insert position of FgMet3 upstream fragment |
| P12 | AGGACATATCCACGCCCTCCTA |
| P13 | TTCCGGAGGACAGACACATCGA | PCR primers to verify the insert position of FgMet3 downstream fragment |
| P14 | GGTTGTACTGTAGAAGGTG |
| P15 | GATCGGTTAAGATGAGA | PCR primers to amplify FgMet14 upstream fragment |
| P16 | CCTTCAATATCATCTTCTGTT  GTGTATATATAAAATTGATG |
| P17 | GGAGACAATACCGGAAGGAAA  TATGTAAATGATGATGATG | PCR primers to amplify FgMet14 downstream fragment |
| P18 | GTACTTGCTGTACCTGGAG |
| P19 | TAATCATATAGAGAGAGCGC | PCR primers to amplify FgMet14 deletion fragment |
| P20 | TTCTTGTTCAGGATTCCGA |
| P21 | ATGGCTACGTAAGTTTGTCC | PCR primers to verify the target fragment of FgMet14 |
| P22 | CTCAGGAGCCTCCATCGAAT |
| P23 | CACATGAAGATGCCTGCAT | PCR primers to verify the insert position of FgMet14 upstream fragment |
| P24 | AGGACATATCCACGCCCTCCTA |
| P25 | TTCCGGAGGACAGACACATCGA | PCR primers to verify the insert position of FgMet14 downstream fragment |
| P26 | GATGATGCCGTCTCGTCTCTG |
